# Strontium‐Enriched Barite for Enhanced PbSO_4_ Nucleation in Lead‐Acid Batteries

**DOI:** 10.1002/smll.202409902

**Published:** 2025-03-03

**Authors:** Colin T. Campbell, Ajay S. Karakoti, Shannon J. Lee, Carinna Lapson, David Reed, Benjamin A. Legg, Vijayakumar Murugesan

**Affiliations:** ^1^ Pacific Northwest National Laboratory Richland WA 99354 USA; ^2^ Department of Chemistry and Biochemistry, University of Oregon Eugene OR 97403 USA

**Keywords:** epitaxy, in situ AFM, nucleation, Pb‐acid battery, sulfation

## Abstract

Barite (BaSO_4_) crystals are an important additive in lead acid batteries due to their ability to promote the controlled nucleation and growth of anglesite (PbSO_4_) during battery cycling. This is due to BaSO_4_’s isostructural match with PbSO_4_, which facilitates epitaxial nucleation. However, BaSO_4_’s efficacy as a nucleation promoter is limited by lattice mismatches between the two phases of 1–5%. Here, strontium‐enriched barite, (Ba,Sr)SO_4_, is synthesized to produce a nucleating promoter that displays an improved lattice match with PbSO_4_. Nucleation of PbSO_4_ from Pb‐rich sulfuric acid solutions on strontium‐free and strontium‐enriched barite (up to 17 mol% Sr) is investigated using in‐situ atomic force microscopy and optical microscopy. At low strontium concentrations, PbSO_4_ forms by the Stranski–Krastanov growth pathway (monolayer + island formation), with most nucleation occurring on edges and surface defects. At higher concentrations, the layer‐by‐layer growth mechanism known as Frank van‐der‐Merwe growth is observed, which allows for facile growth of continuous PbSO_4_ films across the BaSO_4_ surface. These films are successfully re‐dissolved on exposure to Pb‐free sulfuric acid. The results show that strontium incorporation fundamentally alters the mechanism of PbSO_4_ nucleation on BaSO_4_, and thus provides a potential avenue for creating improved nucleation promoters in lead‐acid batteries.

## Graphical abstract

Barite (BaSO4) particles are commonly added to lead acid batteries, where they facilitate the nucleation and growth of PbSO4 during battery cycling. However, barite’s effectiveness at promoting nucleation is limited by a relatively large lattice‐parameter mismatch with PbSO4. Here, we show that incorporating strontium during barite synthesis can reduce the lattice parameter mismatch and enable a much facile nucleation mechanism.

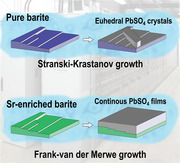

## 1 Introduction

Lead‐acid batteries present a cost‐competitive technology for meeting grid‐scale energy storage needs, but improvements in battery cycle life are needed.^[^ ^1^, ^2^, ^3^ ^]^ The operation of lead‐acid batteries depends on the reversible nucleation, growth, and dissolution of anglesite (PbSO_4_) crystals. Lead‐acid batteries encounter diverse failure modes, which include corrosion of the positive grid, shedding of positive active material, and irreversible sulfation.^[^ ^4^, ^5^, ^6^, ^7^, ^8^ ^]^ Sulfation refers to the accumulation of recalcitrant PbSO_4_ deposits (such as large PbSO_4_ crystals) that resist dissolution^[^ ^9^ ^]^ and reduce the utilization of active material. It is sometimes called “hard sulfation” to distinguish it from the desired formation of PbSO_4_ during regular operation.^[^ ^10^ ^]^ Sulfation often occurs after extended service life, after deep depth of discharge, or in cases where a battery is left partially charged for extended periods of time.^[^ ^2^, ^10^ ^]^ All three scenarios (long service life, deep depth of discharge, and extended rest periods at a partially charged state) are likely to occur in grid‐scale energy storage applications, making it important to create batteries that resist sulfation. Diverse additives have been investigated for controlling sulfation, including lignosulfonates,^[^ ^11^ ^]^ carbon,^[^ ^12^, ^13^, ^14^ ^]^ or phosphoric acid.^[^ ^15^ ^]^ However, barite (BaSO_4_) crystals have historically been one of the most important additives.^[^ ^11^ ^]^

Barite is commonly added to the negative (Pb‐metal) electrode of, due to its ability to prevent sulfation by facilitating the nucleation, growth, and dissolution of PbSO_4_. (It is avoided on the positive electrode, where it can have detrimental effects, such as promoting shedding of the PbO_2_ active material^[^ ^16^ ^]^). It may be added as naturally occurring particles (called barytes), or synthetic (called blanc fixe).^[^ ^17^ ^]^ Because BaSO_4_ is isostructural to PbSO_4_, it templates the formation of PbSO_4_, and reduces the barrier to PbSO_4_ nucleation.^[^ ^9^ ^]^ This enables the repeatable formation of small, more uniformly distributed PbSO_4_ crystals in a Pb‐acid battery that can be easily redissolved during battery charging.^[^ ^18^, ^19^ ^]^ Regarding nucleation in batteries, a reduction of nucleation barrier on lithium metal batteries (expressed as nucleation overpotential^[^ ^9^, ^20^ ^]^) was noted to occur if the substrate (here: BaSO_4_) is miscible in the deposited material (here: PbSO_4_).^[^ ^20^ ^]^ The behavior in that work was credited to a “buffer layer” on the exposed surface of the substrate that has the structure of the deposited material, and continuously approaches the composition of the phase to be deposited (Li in that work, PbSO_4_ in this one). This property of miscibility of the substrate material in the deposited phase is true for the PbSO_4_/BaSO_4_ system in the range of ≈10 mol% Ba or lower.^[^ ^21^, ^22^ ^]^ It is well known that low lattice misfits are important for achieving facile heterogeneous nucleation and growth.^[^ ^23^, ^24^ ^]^ However, our recent work has shown that while surface films of mixed composition appear to form easily, barite's effectiveness as a nucleator of bulk PbSO_4_ is limited by its large lattice misfit with anglesite.^[^ ^25^ ^]^ This study presents a novel approach for creating more effective nucleation promoters: by systematically altering barite's lattice parameters through the incorporation of strontium.

Our work builds on past research on sulfate nucleation and growth, which has been studied for reasons that include Pb‐acid batteries,^[^ ^18^, ^25^ ^]^ paleoclimate modeling,^[^ ^26^ ^]^ environmental distribution of toxic elements,^[^ ^27^ ^]^ scale formation during petroleum extraction,^[^ ^28^, ^29^ ^]^ and fundamental materials science.^[^ ^30^ ^]^ The nucleation of PbSO_4_ on barite is an example of heteroepitaxial nucleation, which is controlled by the interplay of interfacial tensions (including the substrate‐solution, substrate‐precipitate, and precipitate‐solution interfaces) with the epitaxial strain that arises from lattice mismatches between the substrate and the precipitate. Our previous work has shown that PbSO_4_ nucleation on BaSO_4_ occurs via a Stranski–Krastanov (SK) growth mode, in which PbSO_4_ first forms an epitaxial monolayer but subsequent layers grow as discrete islands.^[^ ^25^ ^]^ Formation of PbSO_4_ or mixed (Pb,Ba)SO_4_ epitaxial monolayers can be understood to be promoted by the lower interfacial energy of the PbSO_4_‐solution interface relative to BaSO_4_, or by the stabilization of the surface due to entropy of mixing.^[^ ^25^ ^]^ This free energy difference is significant enough that Pb‐rich monolayers can even grow from solutions that are undersaturated with respect to bulk PbSO_4_.^[^ ^25^ ^]^ However, the PbSO_4_ films are highly strained to match the underlying substrate: the lattice mismatch between BaSO_4_ and PbSO_4_ is roughly 4.7%, 1.1%, and 2.8%, respective to the a, b, and c lattice vectors (following the *Pnma* lattice convention).^[^ ^31^ ^]^ This epitaxial strain suppresses the growth of additional PbSO_4_ layers beyond a single monolayer. To partially relieve this strain energy, growth of additional layers occurs as discrete islands, rather than continuous sheets.^[^ ^32^ ^]^ These islands have a characteristic width that depends on the balance of interfacial tension and elastic strain energy, with larger strain leading to smaller widths.^[^ ^33^, ^34^ ^]^ However, theory predicts that a modest reduction in the lattice mismatch can reduce the epitaxial strain energy, so that multilayer films can form as continuous sheets. This should allow the PbSO_4_ to grow by a more facile layer‐by‐layer mechanism known as Frank‐van der Merwe (FvdM) growth.^[^ ^34^, ^35^ ^]^

In this study, we propose that we can create improved nucleation‐promoters by synthesizing crystals with a better lattice match to PbSO_4_. For example, the mineral celestite (SrSO_4_) is also isostructural to anglesite, and displays a better lattice match with anglesite than barite. Previous work shows that PbSO_4_ grows on SrSO_4_ via the FvdM growth mode.^[^ ^15^ ^]^ SrSO_4_ has previously been considered as a nucleating agent in Pb‐acid batteries, but pure SrSO_4_ has drawbacks that limit its use.^[^ ^36^ ^]^ BaSO_4_ has a relatively low solubility (K_sp_ = 1.142·10^−10^ at 25 °C)^[^ ^37^ ^]^ that makes it stable during battery operation, but SrSO_4_’s solubility (K_sp_ = 2.409·10^−7^ at 25 °C)^[^ ^37^ ^]^ is even higher than PbSO_4_ (K_sp_ = 1.72·10^−8^ at 25 °C).^[^ ^38^ ^]^ Sr has a tendency to incorporate into PbSO_4_ precipitates,^[^ ^39^ ^]^ and there concerns about the ability of PbSO_4_ nucleated on SrSO_4_ to redissolve.^[^ ^36^ ^]^ However, solid solutions of (Ba,Sr)SO_4_ are possible,^[^ ^40^, ^41^, ^42^, ^43^ ^]^ and should retain some of BaSO_4_’s desired low solubility while providing an improved lattice match with PbSO_4_^[^ ^40^, ^44^ ^]^ that could dramatically enhance nucleation by changing the mechanism from SK growth to FvdM growth.^[^ ^35^ ^]^ If this leads to more uniform and/or well‐adhered nuclei of PbSO_4_, it may also reduce phenomena such as particle‐shedding or Ostwald ripening, which are known to drive sulfation and degrade battery performance.^[^ ^8^, ^45^, ^46^ ^]^ To test this hypothesis, we have synthesized a set of (Ba,Sr)SO_4_ crystals with varied strontium incorporation: pristine (0 mol% Sr), lightly enriched (6 mol% Sr), and highly enriched (17 mol% Sr). These samples are henceforth referred to as 0%Sr, 6%Sr, and 17%Sr, respectively. We then used a combination of in situ and ex situ microscopies to observe the resulting changes in nucleation mechanism.

## 2 Characterization of Sr‐Enriched BaSO_4_

### 2.1 Crystal Morphology

**Figure 1:**
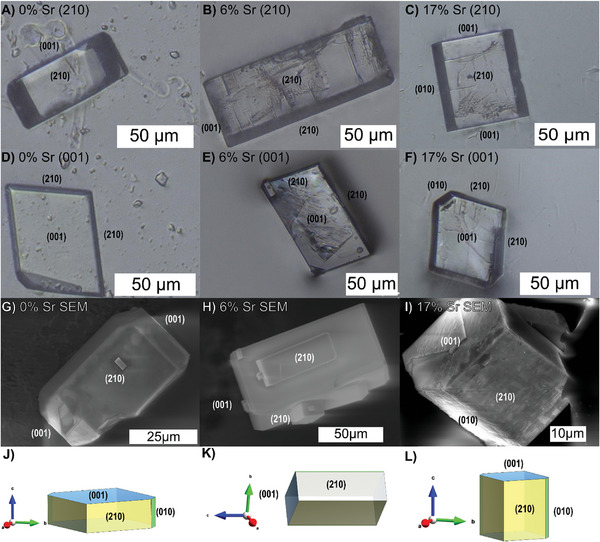
(A–C) show top‐down optical microscopy views of a (210) surface for 0%Sr, 6%Sr, and 17%Sr crystals, respectively. (D–F) show top‐down optical microscopy views of a (001) surface for 0%Sr, 6%Sr, and 17%Sr crystals, respectively. (G–I) show SEM images of the 0%Sr, 6%Sr, and 17%Sr crystals, respectively. Rectangular faces are typical of the (210) surfaces. Rhombic faces are typical of the (001) surfaces. (J–L) show illustrations of crystal forms observed in subfigures (A–C), highlighting the tabular nature of the 0%Sr crystal, the elongated form of the 6%Sr crystal, and the intermediate nature of the 17%Sr crystal. The {001} and {210} faces are dominant, and the {010} facets are minor surfaces.

The morphology of (Ba,Sr)SO_4_ crystals depends on synthesis conditions (i.e., temperature, saturation, ionic strength, and Sr concentration).^[^ ^29^, ^47^ ^]^ Our syntheses (0%Sr, 6%Sr, and 17%Sr) all produced micron‐scale euhedral crystals (**Figure** **1**). The crystals show well‐developed rectangular facets (Figure 1A–C) and rhombic facets (Figure 1D–F), which were identified as the {210} and {001} faces, respectively, based on their morphology and atomic‐resolution atomic force microscopy (AFM) imaging. The particle shapes ranged from tabular crystals dominated by the {001} faces to elongated crystals dominated by the {210} faces. Additional minor facets, such as {010}, are also observed via secondary electron microscopy (SEM) (Figure 1G–I). There was some variation within each synthesis, and between syntheses. For example, 0%Sr showed both tabular forms (Figure 1 A,D) and elongated forms (see Figure S1, Supporting Information). The 6%Sr synthesis produced similar morphologies, but the crystals tended to be more elongated in the [001] direction and had narrower minor facets. The 17%Sr crystals were less elongated in the [001] direction and slightly smaller in size than the 6%Sr crystals but tended to express the {010} facets more strongly. In summary, the three syntheses produced particles of similar size, and shape. All particles were dominated by the {210} and {001} faces, but the Sr‐enriched particles tended to be more elongated in the [001] direction and had a slightly greater tendency to express the {010} facets.

### 2.2 Sample Composition and Lattice Parameters

#### 2.2.1 Single Crystal X‐ray diffraction (XRD)

Single crystal XRD was performed to determine the lattice parameters of crystals from each synthesis. Complementary inductively coupled plasma mass spectrometry (ICP‐MS) analysis of each synthesis was performed to establish a relation between the Sr content in BaSO_4_ and the lattice parameter. Supporting energy dispersive X‐ray spectroscopy (EDS) measurements that illustrate the uniformity of Sr‐incorporation are shown in Figures S2–S4 (Supporting Information). Single crystal XRD‐derived lattice constants and related calculations are shown in **Table** **1**. We observe a roughly linear relation between (Sr,Ba)SO_4_ lattice parameter and Sr concentrations for the a and c parameters, consistent with Vegard's law and existing literature,^[^ ^31^, ^43^, ^48^, ^49^, ^50^ ^]^ but the b parameter was insensitive to Sr‐content (Figure S5, Supporting Information). The limited change of the b‐parameter was not expected, but positive deviations from Vegard's law have previously been reported for this parameter in (Ba,Sr)SO_4_ crystals.^[^ ^43^, ^44^ ^]^ The lattice misfit with PbSO_4_ was estimated by comparison with lattice parameters reported by Miyake et. al. (a = 8.842 Å, b = 5.398 Å, and c = 6.959 Å, using the *Pnma* lattice convention).^[^ ^31^ ^]^ In all cases, the misfits are largest in the [100] direction and smallest in the [010] direction.

**Table 1:** Lattice parameters of representative (Ba,Sr)SO_4_ crystals, as measured using single crystal XRD. Parameter refinement and errors obtained using the APEX5 software (Bruker). Additional calculations of percent lattice misfit relative to PbSO_4_, and estimates of the elastic strain energy required to form an epitaxial film of PbSO_4_ on the (100), (010), and (001), and (210) faces (in kJ mol^−1^) are also provided.

	a (Å) [100]	b (Å) [010]	c (Å) [001]	U(100)	U(010)	U(001)	U(210)
				(kJ mol^−1^)			
0%Sr	8.8860(5) +4.8%	5.4555(3) +1.1%	7.1598(4) +2.9%	2.193	5.429	5.539	3.474
6%Sr	8.865(2) +4.5%	5.4575(9) +1.1%	7.149(1) +2.7%	2.002	5.321	5.092	3.171
17%Sr	8.7997(4) +3.7%	5.4534(2) +1.0%	7.1157(3) +2.3%	1.405	3.645	3.622	2.224

#### 2.2.2 Strain Energy Calculations

Based on the estimated lattice mismatch, elastic strain energies for epitaxial PbSO_4_ films grown on the (001), (010), (100), and (210) faces were estimated. The calculations assume that the PbSO_4_ film is strained in‐plane to be commensurate with the substrate, with zero out‐of‐plane stress conditions.^[^ ^51^, ^52^ ^]^ In the absence of literature values for PbSO_4_ elastic constants, the elastic stiffness tensor for BaSO_4_ was used.^[^ ^53^ ^]^ For the (210) surface, coordinate transforms were performed following Grundmann.^[^ ^54^ ^]^ The results of these calculations are presented in Table 1. Because the strain energy is roughly parabolic in the lattice mismatch, the strain energies on the 17%Sr surfaces are predicted to be drastically reduced relative to those on 0%Sr. We estimate a reduction of roughly 35% for the (001) surface, and 36% for the (210) surface.

#### 2.2.3 Powder XRD

Powder XRD (Figure S6, Supporting Information), provides insight on sample uniformity. The 0%Sr and 6%Sr showed relatively sharp peaks, with the 6%Sr peaks shifting to higher 2θ, reflecting a reduction in lattice parameters. The 17%Sr sample was shifted further still, and also showed noticeable peak broadening, with a tail toward higher 2θ. Similar broadening has been observed in literature (Ba,Sr)SO_4_ synthesis.^[^ ^55^ ^]^ This is likely due internal compositional variations due to zonation, or growth of particles with varied concentration. It is possible that Sr‐concentration increases at later stages of particle synthesis, as the Ba concentration becomes increasingly depleted. However, there are no additional peaks that would occur of distinct endmember phases (like pure SrSO_4_) were present.

### 2.3 Surface Morphology and Stability of (Ba,Sr)SO_4_ Particle Surfaces

**Figure 2:**
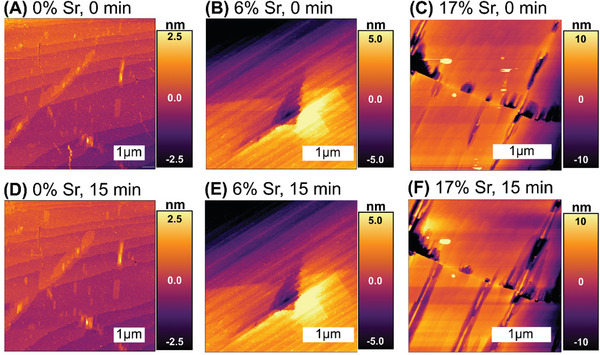
(A–C) show initial AFM topography images a (210) surface for 0%Sr, 6%Sr, and 17%Sr surfaces, respectively, obtained immediately after loading in the cell. The 0%Sr surface shows a broad step‐and‐terrace morphology, with some hillocks and small pits. The 6%Sr surface shows a narrower step‐and‐terrace morphology, with some surface defects. The 17%Sr surface shows significant pits on the order of 10 nm in depth. (D–F) show the change in morphology after 15 min of exposure to flowing 100 mm H_2_SO_4._ The 0%Sr and 6%Sr surfaces show insignificant change, but the pits on the 17%Sr sample have become noticeably broader and deeper.

To check the stability of the crystals in sulfuric‐acid conditions, similar to those observed in lead‐acid batteries, we performed in situ AFM imaging immediately after exposure to 100 mM H_2_SO_4_, and then after 10 min of exposure to 100 mM H_2_SO_4_, actively flowing at 0.1 mL min^−1^ (**Figure** **2**). AFM images of the (210) surface of 0%Sr crystals and 6%Sr crystals showed step and terrace morphologies, and showed little change between the initial images and those obtained after 15 min of exposure to flowing H_2_SO_4_ solution. The resistance to change is consistent with pristine BaSO_4_’s low solubility. However, the 17%Sr crystals had an initially pitted surface, with pits on the order of ten nm deep. These pits became noticeably deeper and wider during exposure to flowing H_2_SO_4_ solution. The increased tendency to dissolve is consistent with SrSO_4_’s higher solubility than BaSO_4_, and the debated non‐ideality of (Sr,Ba)SO_4_ solid solutions.^[^ ^41^, ^44^, ^54^, ^56^ ^]^

### 2.4 Solubility of (Ba,Sr)SO_4_ Particles

For barite to persist in a battery, it must resist dissolution. The solubility of (Ba,Sr)SO_4_ particles has been studied extensively,^[^ ^41^, ^44^, ^55^, ^56^, ^57^, ^58^, ^59^, ^60^ ^]^ but is still not fully understood. (Ba,Sr)SO_4_ is commonly approximated as a regular solution, but its dissolution is a prototypical example of a solid‐solution aqueous‐solution (SSAS) process that is much more complex than simple dissolution of pure phases like BaSO_4_ or SrSO_4_.^[^ ^61^ ^]^ Its dissolution is often non‐congruent (i.e., non‐stoichiometric), and its composition can evolve slowly for years.^[^ ^59^ ^]^ It is likely that barite dissolves roughly congruently until the solution becomes saturated with respect to pure barite. Subsequent dissolution will release Sr into solution, while Ba^2+^ will be sequestered into the pure barite. Analysis is made more complex by the ability of barite interfaces to form impurity‐enriched layers,^[^ ^25^, ^62^ ^]^ with earlier work indicating that Sr‐enriched surfaces may occur.^[^ ^59^ ^]^ These processes are reflected in the complex nature of the surface dissolution observed for 17%Sr. Regardless, most studies conclude that (Ba,Sr)SO_4_ particles will release less Sr into solution than pure Sr, reaching concentrations that scale with Sr‐content of the particles.^[^ ^31^ ^]^

To assess the stability of our particles in battery‐relevant conditions, we measured their dissolution into 4.5 m sulfuric acid at 50 °C. ≈40 mg were added to 21 mL of acid and equilibrated for several days. We observed non‐congruent dissolution, with preferential release of Sr relative to Ba (see Table S1, Supporting Information). Ba concentrations in the acid phase reached maximum concentrations of 0.014 mg L^−1^, just above the detectable limits. Sr reached higher concentrations. The 17%Sr sample released 1.56 mg L^−1^ Sr after 90 h of dissolution and stabilized there, only increasing to 1.58 mg L^−1^ at 180 h. But even this is less than 2% of the total Sr present in the solid particles, indicating that the particles are relatively stable.

## 3 Nucleation and Growth of PbSO_4_ on (Ba,Sr)SO_4_ Particle Surfaces

### 3.1 In Situ AFM Observations on (Ba,Sr)SO_4_ (210)

After initial characterization of the crystal surfaces, subsequent time‐resolved AFM imaging was performed to examine the mechanism of PbSO_4_ nucleation and growth. Growth was induced by flowing a solution containing 100 mm H_2_SO_4_ and 50 µm Pb(NO_3_)_2_ that is supersaturated with respect to PbSO_4_ (saturation index = 0.501, using log_10_ scale as calculated with Visual MINTEQ 3.1).^[^ ^63^ ^]^ This saturation is sufficient to produce multilayer film growth without creating significant homogeneous nucleation in solution.

**Figure 3:**
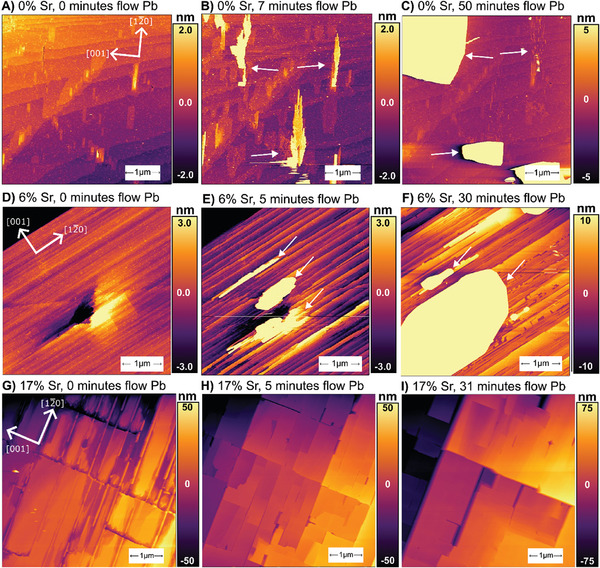
(A–C) show the topography of a 0%Sr (210) surface. (A) Prior to introduction of Pb‐containing solution we observe a step‐and‐terrace morphology, with some hillocks and pits. (B) After 7 min of exposure to Pb‐solution, we observe growth of nanometer‐height islands, elongated in the [1$\bar{2}$0] direction. These appear to have nucleated on preexisting hillocks and pits. (C) After 50 min of exposure to Pb‐solution, many of the nanometer‐height islands have redissolved and we see formation of micron‐scale crystals. (D–F) show topography of 6%Sr (210) surface. (D) Prior to introduction of Pb‐containing solution we observe a step and terrace morphology, with a prominent pit and hillock. (E) After 5 min of exposure to Pb‐solution, we observe nm‐scale deposits, elongated in the [1$\bar{2}$0] direction, the largest of which appear to have nucleated from the pit and hillock. (F) After 30 min exposure to Pb‐solution, we see these growths have ripened into micron scale crystals. (G–I) shows the topography of the 17%Sr surface. (G) Prior to introduction of Pb‐containing solution we see a surface with network of elongated pits, ≈10 nm deep. (H) After 5 min exposure to Pb‐solution, the pits have filled into produce flat terraces, several hundred nm in width. (I) After 31 min exposure to Pb‐solution, the surface shows continued growth of layers of PbSO_4_, creating increasingly flat terraces. No nucleation of large crystals is observed for samples at this Sr content.

Growth on the (210) BaSO_4_ surface is documented in **Figure** **3**. On 0%Sr, the initial surface shows a typical step and terrace morphology with a few small hillocks between 1 and 2 nm in height (Figure 3A). Immediately after exposure to Pb‐containing solution, a monolayer of PbSO_4_ nucleates from step‐edges and rapidly propagates across the surface. Next, multilayer precipitates ≈3 nm thick are seen to nucleate at some of the larger hillocks (Figure 3B). The largest of these features are highly elongated along the [1,0] $\bar{2}$ direction (see Figure S7, Supporting Information for high‐resolution lattice images processed using the “Gwyddion” software package).^[^ ^64^ ^]^ Based on their limited thickness and their alignment along the substrate's lattice, we conclude that these are epitaxial growths. Higher resolution images (Figure S8, Supporting Information) show that many of the islands are subdivided into “fingers” ≈80 nm wide. This is an indication of SK‐type growth, where island width is limited by the epitaxial strain energy.^[^ ^33^, ^65^ ^]^ Here, we suspect that the elongation is driven by a lower lattice mismatch in the [1,0] $\bar{2}$ direction (2.5%) than in the [001] direction (2.9%), and the stiffer elastic constant in the [001] direction (103.8 GPa stiffness for the BaSO_4_ [001] direction, versus 89.3 GPa for the BaSO_4_ [1,0] direction $\bar{2}$.^[^ ^31^, ^54^ ^]^ However, these nm‐height islands are short‐lived. Even under continuous flow of supersaturated solution, they only last for a few minutes and then quickly redissolve, concurrent with the appearance of larger PbSO_4_ crystals that exceed hundred nanometers in height (Figure 3C). Presumably, the intrinsic epitaxial strain of the islands makes them less stable than the larger PbSO_4_ crystals (which have previously been found to have lattice parameters characteristic of bulk PbSO_4_).^[^ ^15^ ^]^ Thus, the nm‐thickness islands only exist transiently before transforming into larger, more stable PbSO_4_ crystals or by being consumed by larger PbSO_4_ crystals growing nearby.

The nucleation behavior of PbSO_4_ on 6%Sr (Figure 3D–F) is qualitatively the same as that for 0%Sr. The (210) surface shows a step‐and‐terrace morphology, although the step widths and orientations are different than for 0%Sr. Transient epitaxial features grow first and are later consumed by large crystals. These transient features are elongated in the [1$\bar{2}$0] direction, but they are wider in the [001] direction than before, with some reaching ≈300 nm in width. Although the width may be influenced by interactions with the terraces, the increase likely reflects a reduction in strain energy relative to 0%Sr.

A significantly different behavior is observed for nucleation on 17%Sr crystals. The initial (210) surface shows a more pitted morphology (Figure 3G), due to the dissolution described in Section 2.3. On exposure to the lead‐containing solution, PbSO_4_ grows from the surface in a layer‐by‐layer extension of the existing substrate. The pits fill in to produce a film ≈150 nm thick, composed of flat rectangular (210) facets. These surfaces grow in a layer‐by‐layer manner and merge together. This layer‐by‐layer growth of continuous films marks a transition to FvdM growth, which is consistent with a significant reduction in lattice mismatch.

### 3.2 In Situ AFM Observations on (Ba,Sr)SO_4_ (001)

**Figure 4:**
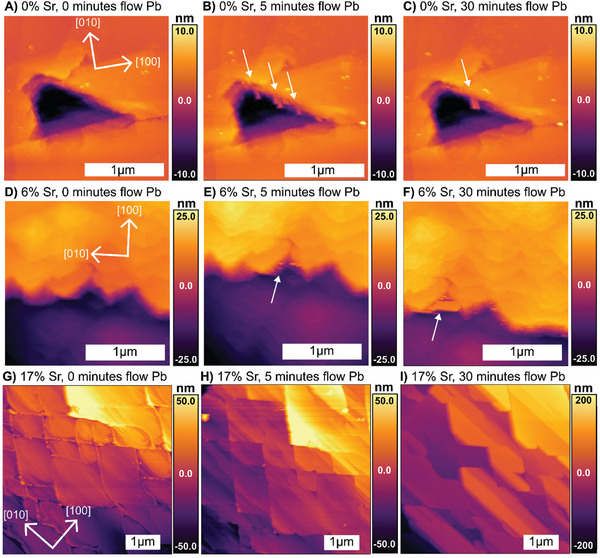
(A–C) shows formation of transient euhedral crystals on the (001) surface of a 0%Sr particle, with initial image showing a large pit prior to Pb‐exposure, and subsequent images showing the nucleation of multiple euhedral PbSO_4_ crystals in the defect after 5 min, and the coarsening of these crystals after 30 min. (D–F) shows formation of transient euhedral crystals on the (001) surface of a 6%Sr particle. The initial image shows a step‐and terrace morphology with hillocks and some large (several‐nm high) steps. Subsequent images show the formation of multiple euhedral crystals on the larger steps after 5 min of Pb‐exposure and their coarsening to produce a large (≈200 nm scale) crystallite after 30 min. (G–I) shows formation of transient euhedral crystals on the (001) surface of a 17%Sr particle. The initial image shows rhombic terraces with signs of etching at their edges. Subsequent images show layer‐by‐layer growth during Pb‐exposure, to produce progressively smoother (001) facets.

Nucleation of PbSO_4_ on (001) surfaces (**Figure** **4**) shows a similar dependance on Sr content as was observed for the (210) surfaces. The (001) surfaces examined here show varied morphologies prior to exposure to Pb‐rich solution. The 0%Sr (001) surface showed a mixture of terraces and deep pits, the 6%Sr (001) surface shows a mixture of terraces and growth hillocks, and the 17%Sr (001) surface displays pattern of rhombic facets, with signs of etching at their edges.

After exposure to Pb‐containing solution, the 0%Sr and 6%Sr surfaces both produce transient epitaxial features. However, the growths appear more akin to euhedral crystals than the ≈3 nm thick films that were observed on the [210] surface: their morphology shows crystal faceting and strong elongation in the [010] direction. But they do not appear to be as thermodynamically stable as bulk PbSO_4_: they undergo rapid coarsening, and the smaller crystals quickly dissolve in favor of progressively larger crystals. Moreover, they never appear directly on the [001] surface, they only form on large surface defects (defects many nm in height) that appear to template their nucleation. Further examples of growths on the 0%Sr and 6%Sr (001) surface are shown in Figure S9 (Supporting Information). However, nucleation on the (001) surface of 17%Sr is drastically different than on the lower‐strontium samples. Rather than nucleating crystals at surface defects, layer‐by‐layer growth occurs across the entire surface to create a terraced surface with facets that become progressively smoother over time. Thus, the transition from SK to FvdM growth appears to occur on both major barite surfaces.

### 3.3 Ex Situ Observation of Grown PbSO_4_

**Figure 5:**
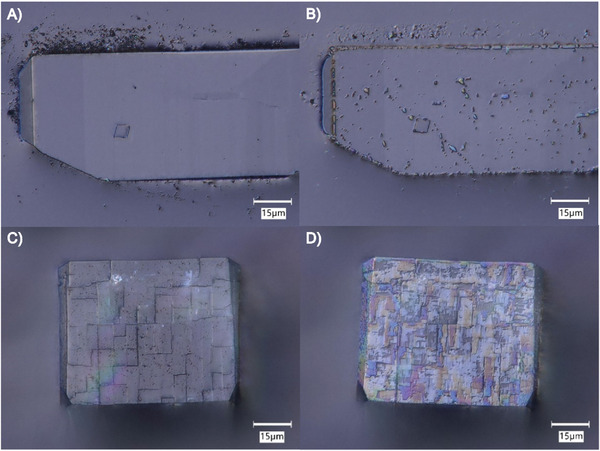
(A) Optical image of a pristine 0%Sr crystal, focused on the (210) surface, shows a euhedral crystal with flat surfaces. (B) The same crystal after 24 h of exposure to 100 µM Pb(NO_3_)_2_ and 100 mm H_2_SO_4_ solution shows a distribution of micron‐scale deposits across the surface, with especially heavy growth at the edges of facets. (C) Optical image of a pristine 17%Sr crystal, focused on the (210) surface, shows a euhedral crystal with numerous optically visible terraces on the exposed surfaces. (D) The same crystal after 24 h of exposure to 100 µm Pb(NO_3_)_2_ and 100 mm H_2_SO_4_ shows a patchy iridescent surface, with no evidence of discrete deposits, indicating the formation of continuous PbSO_4_ films.

The Cypher AFM used for this study has a limited scan range that cannot image the entire BaSO_4_ crystal. Thus, complementary ex situ optical imaging was used to determine the distribution of precipitates across a barite crystal. **Figure** **5** shows 0%Sr and 17%Sr samples before and after 24 h of exposure to a solution containing 100 µm Pb(NO_3_)_2_ and 100 mm in H_2_SO_4_. Results for the 6%Sr samples are shown in Figure S10 (Supporting Information), and appear similar to the 0%Sr sample.

The images show that 0%Sr fosters nucleation and growth of micron‐scale PbSO_4_ crystallites, scattered across the surfaces. PbSO_4_ preferentially forms along the edges of the crystal, which is consistent with the AFM observation that nucleation tends to occur on multi‐step topographic features (edges are likely to be rich in complex topographical features). Such discrete growths are completely absent from the 17%Sr sample (Figure 5D). Instead, we see an iridescent pattern covering most of the surface that indicates the formation of PbSO_4_ with thickness on the order of optical wavelengths (i.e., hundreds of nanometers). The patches tend to follow the rectangular pattern of the terraces, similar to what was observed on the (210) surface in AFM. Thus, optical imaging confirms that high levels of strontium enrichment trigger a transition from nucleation of distinct PbSO_4_ islands to a layer‐by‐layer growth mode. Images of the (001) surface also show this transition (Figure S11, Supporting Information). Moreover, the layer‐by‐layer grown films seem to be sufficiently stable that they are not transformed into euhedral crystals like those seen on the 0%Sr, even after hours of aging. The epitaxial films formed by PbSO_4_ on Sr‐rich substrates are in intimate crystallographic contact and would be unlikely to undergo any sort of mechanical shedding (as might potentially happen for the discrete particles that form on particles of lower Sr content).

## 4 Dissolution of Grown PbSO_4_

**Figure 6:**
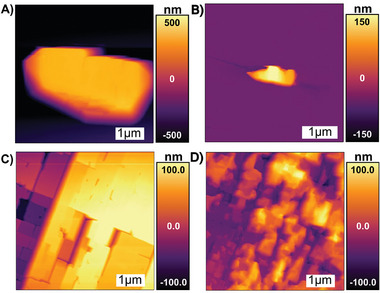
(A) shows a large euhedral PbSO_4_ crystal grown on the 0%Sr (210) face, after 50 min of exposure to a supersaturated Pb‐containing solution. (B) shows the same particle after 50 min exposure to a flowing Pb‐free H_2_SO_4_ solution. The particle has significantly dissolved, and the underlying defect that nucleated particle formation is clearly visible. (C) shows a 17%Sr (210) surface, with terraced rectangular facets formed by 50 min of exposure to a supersaturated Pb‐containing solution. (D) shows the same surface after 50 min of flow of Pb‐free H_2_SO_4_ solution, which leaves behind a patchy network of deposits but largely re‐exposes the substrate's initial topography (compare with Figure 3G).

For a Pb‐acid battery to last over many cycles, PbSO_4_ must be deposited and removed facilely. Thus, the ability redissolve these films is of key importance. Past studies have suggested that PbSO_4_ grown on SrSO_4_ may be more difficult to redissolve than those grown on BaSO_4_.^[^ ^36^ ^]^ Thus, after growing PbSO_4_, we conducted additional experiments to confirm whether the PbSO_4_ grown on these surfaces could be redissolved in a simulated charging process. In these experiments, the samples were exposed to a flowing solution of Pb‐free 100 mm sulfuric acid. These results are shown in **Figure** **6**.

The 0%Sr sample initially shows a single large, ≈4 µm long PbSO_4_ crystal. After 50 min exposure to Pb‐free 100 mm sulfuric acid, the crystal has shrunk to less than 1 µm in length. The local topographic features of the BaSO_4_ surface, which presumably fostered its initial nucleation and growth, are clearly visible. As discussed previously, the 17%Sr sample shows no such large PbSO_4_ crystals, but instead showed a uniform PbSO_4_ film, which then underwent significant dissolution when exposed to Pb‐free solution. The film decomposed into small islands, and ultimately left a pattern of hillocks, exposing some (but not all) of the original surface that was present before film growth. In both cases, most of the deposit was successfully redissolved without significant damage to the underlying substrate. This shows clearly that (1) when PbSO_4_ is grown on the (Ba,Sr)SO_4_ substrate it retains its ability to be redissolved (which is critical for battery cycling), and also shows that the (Ba,Sr)SO_4_ substrate is significantly more resistant to dissolution than the PbSO_4_ (which is essential if the particles are to persist throughout the life of the battery). Although the detailed kinetics of dissolution in battery‐relevant conditions remain to be studied, these initial results show that it is, in principle, possible to redissolve the PbSO_4_ growths from a Sr‐substituted BaSO_4_ while retaining the nucleation‐promoting substrate.

## 5 Conclusion

**Figure 7:**
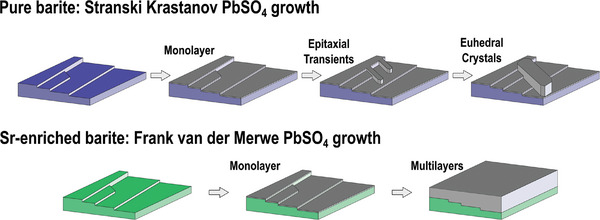
Illustration of the observed effect of Sr incorporation into BaSO_4_ on PbSO_4_ growth. Pure BaSO_4_ has a poor lattice match, resulting an SK‐growth mode of rapid monolayer formation, followed by formation of nanoscale epitaxial transient features at surface‐defects, followed by eventual nucleation of euhedral crystals. In contrast, high enrichments of Sr provides a better lattice match PbSO_4_, to the more facile layer‐by‐layer FvdM growth mode, in which monolayer formation is followed by layer‐by‐layer growth to form continuous films.

We have successfully synthesized strontium‐substituted (Ba,Sr)SO_4_ with concentrations up to 17 mol%, to obtain a solid‐solution with lattice parameters that more closely match those of PbSO_4_. This Sr‐substituted barite does more than provide enhanced nucleation rates, it fundamentally alters the mechanism of PbSO_4_ nucleation and growth, leading to broader coverage of the substrate and fewer discrete crystals (for illustration, see **Figure** **7**).

On pristine barite (0%Sr) and lightly Sr‐enriched barite (6%Sr), PbSO_4_ precipitation proceeds through three stages: 1) rapid formation of epitaxial monolayers, 2) elongated nanometer‐scale islands that are several layers thick nucleate and grow from defect sites, and 3) eventual formation of large euhedral crystals with redissolution of the initial nanometer‐scale islands. As discussed in our past work,^[^ ^25^ ^]^ this pathway is a clear example of the SK growth mode, where nucleation is controlled by a competition between interfacial energies and epitaxial strain. The instability of the islands formed in step 2 is believed to reflect the destabilizing influence of epitaxial strain. The elongated morphologies of these islands (with greater elongation in the direction of lower epitaxial strain) further supports this hypothesis. Under this model, the preferential nucleation that we observed defects such as step‐edges on crystal faces and the edges where facets meet is expected, since these are locations where strain energies are partially relaxed. Our results provide new insights into the function and limitations of pure BaSO_4_ as an additive in lead‐acid batteries: its low solubility makes it a stable substrate, and it presents a sufficiently good lattice match that it can promote the nucleation of PbSO_4_, nucleation on pure barite is limited to a small number of defect sites.

However, the highly Sr‐enriched barite (17%Sr) shows a dramatically different nucleation and growth process. On these crystals, PbSO_4_ grows in a layer‐by‐layer process to produce continuous PbSO_4_ films across the entire surface, a clear example of the FvdM growth mode. This is achieved, thanks to a reduced lattice mismatch is estimated to reduce the epitaxial strain energy by ≈32–36% relative to bulk BaSO_4_, which is sufficient to change the nucleation mechanism. The films that form on strontium‐enriched barite are more uniform than the discrete islands found on pristine BaSO_4_, and can also be redissolved without significant damage to the underlying Sr‐substituted BaSO_4_. There is some evidence for enhanced dissolution of the strontium‐rich barite relative to pristine BaSO_4_, which should be investigated in future work, but the Sr‐enriched crystals still show much greater resistance to dissolution than PbSO_4_ or our expectations for pure SrSO_4_, and thus are likely to function as a stable nucleation promoter in battery environments. Our results show that Sr‐substituted barite possess a unique combination of functionality that cannot be achieved by either pure BaSO_4_ or pure SrSO_4_ nucleation promoters. In particular, the ability to change the fundamental nucleation and growth modes from SK growth to FvdM growth may have dramatic influence on battery performance that should be explored in future work. Insofar as nucleation‐control is essential to many battery systems including lithium metal,^[^ ^20^ ^]^ aqueous zinc,^[^ ^66^ ^]^ sodium,^[^ ^67^ ^]^ and Pb‐acid^[^ ^8^, ^9^, ^17^, ^18^, ^19^ ^]^ the strategy shown here for producing more effective nucleation‐promoters via lattice‐parameter engineering may have wide‐ranging applications.

## 6 Experimental Section

### Hydrothermal Synthesis Methods

Pure and Sr‐substituted BaSO_4_ single crystals were synthesized following a protocol modified from Blount.^[^ ^68^ ^]^ All the glassware were rinsed with DI water and dried prior to synthesis. For pure BaSO_4_, 500 mL of an acidified 0.01 m BaCl_2_ solution was made by dissolving BaCl_2_·2H_2_O into 0.3 m HCl and transferred to a 1 L flask. The flask was placed in an oven and brought to 90 °C. At this temperature, 500 mL of 0.01 m Na_2_SO_4_ was slowly added to the flask at 5 mL h^−1^ using a peristaltic pump, over ≈4 days. The sample was filtered using a 0.02 nm PTFE filter while hot. The precipitate was washed with ethanol and water and dried in an oven overnight. Sr‐substituted BaSO_4_ was synthesized following the same protocol and varying the concentration of BaCl_2_·2H_2_O and anhydrous SrCl_2_ to achieve different substitution levels in the sample as indicated in **Table** **2**. The samples hereafter named 6%Sr and 17%Sr had measured atomic percents of Sr of 5.7% and 16.9%, respectively.

**Table 2:** Concentrations of Ba and Sr used in the synthesis of each sample, with measured atomic percent from ICP‐MS. Final significant figure in parenthesis designates estimated uncertainty.

Sample Name	BaCl_2_ Conc. (M)	SrCl_2_ Conc. (M)	Atomic percent Sr
0%Sr	0.01	0	–
6%Sr	0.009	0.001	5.7(0)
17%Sr	0.007	0.020	16.9(1)

### ICP‐MS Analysis

For evaluation of (Ba,Sr,)SO_4_ crystal composition, crystals were dissolved using an alkaline 0.2 m EDTA solution, adjusted to pH 12 using sodium hydroxide. 100 mg of BaSO_4_ or Sr‐substituted barite crystals was added to 25 mL of EDTA solution. The suspension was stirred vigorously at 70 °C for 4 h or until no crystals were visible. The solution was cooled to room temperature and transferred to a pre‐cleaned vial for ICP‐MS analysis using an Agilent Technologies 8900 triple quadrupole ICP‐MS.

For evaluation of dissolution into sulfuric acid, ≈40 mg of (Ba,Sr)SO_4_ crystals were added to 21 mL 4.5 m sulfuric acid. The solution was heated to 50 °C and stirred using a magnetic stirrer at 600 RPM for 90 or 180 h. After dissolution, the sample was cooled and 10 mL of each sample extracted using a 450 nm syringe filter. The solutions were diluted in 2% HNO_3_ (Fisher Scientific Trace metal grade, Assay 67–70%) and analyzed using Agilent 8900 Triple Quadrupole ICP‐MS. Blanks contaning 4.5 m H_2_SO_4_ and DI water were analyzed and showed 0.005 mg L^−1^ or less of Ba and Sr. The instrument was calibrated using the ICP‐MS standards from High‐Purity Standards company to generate the calibration curves, over a range from 0.1 to 100 ppb. Samples were run at several dilutions to bring the elemental concentrations within the instrument's optimal analytical ranges.

### XRD Methods

Unit cell lattice parameters were determined via single crystal X‐ray diffraction (SCXRD) at 300 K using a Bruker D8 Quest with a Mo source (λ = 0.71073 Å). Additional powder X‐ray diffraction (PXRD) was performed on a Rigaku Miniflex 6G benchtop X‐ray diffractometer with D/teX Ultra 0D/1D high‐speed detector with Cu‐Kα radiation source with a Ni‐Kβ filter.

### SEM/EDS Methods

Elemental mapping and analysis were conducted to determine the relative ratios of Sr:Ba, as well as to identify the growth of PbSO_4_ on the surface of the BaSO_4_ crystals. A ThermoFisher Apreo 2 SEM with EDS detector was used. (Ba,Sr)SO_4_ crystals were placed on a conductive carbon adhesive on an aluminum stub and imaged using a 20 kV electron beam. EDS mapping was performed on multiple crystals of each Sr:Ba ratio. To avoid charging, low vacuum mode was used with a chamber pressure of 100 Pa.

### AFM Methods

AFM was performed using a Cypher VRS (Asylum Research, Oxford Instruments) with a liquid perfusion cell and amplitude‐modulated liquid AFM imaging mode with photothermal excitation. The probes used were Arrow‐UHFAuD (NanoWorld). Prior to imaging, the probes were cleaned for ≈5 min in a UV‐ozone cleaner. A PEEK tip clamp and a gold‐reflex coated probe were used to reduce corrosion couples. AFM samples were prepared by mounting a 10 mm grade V1 mica disk onto a steel AFM sample puck using Crystalbond 509 resin. An additional layer of resin was then applied on top of the mica substrate, and heated until soft. BaSO_4_ powder was sprinkled on the hot, flat resin surface, where it adheres, and remains stationary for subsequent measurement. The cooled sample was loaded into the instrument, and a droplet of 100 mm H_2_SO_4_ was placed on its surface. After finding a suitable site for imaging, a high‐resolution image was obtained to image the crystal lattice and confirm which face was being observed. Then a syringe pump (Harvard Apparatus) was used to flow 100 mm H_2_SO_4_ across the surface for 15 min at a flow rate of 0.1 mL min^−1^ while obtaining micron‐scale images. The flow rate was chosen to balance time‐efficient replacement of the solution (which takes ≈2 min at this rate) against the limitations of our AFM (which encounters instabilities for flow rates above 0.2 mL min^−1^). The sample was then allowed to sit for ≈1 h to fully equilibrate with solution. Next, a solution of Pb‐containing sulfuric acid was freshly‐prepared by mixing equal volumes of 200 mm H_2_SO_4_ and 100 µm Pb(NO_3_)_2_, yielding a solution 50 µm in Pb(NO_3_)_2_ and 100 mm in H_2_SO_4_. This solution flowed across the sample at 0.1 mL min^−1^ for ≈50 min, while imaging to observe the growth of PbSO_4_ films and crystals. After the flow of supersaturated solution was concluded, the samples were either exposed to a flow of Pb‐free 100 mm H_2_SO_4_ for an identical 50 min duration at 0.1 mL min^−1^ to image PbSO_4_ dissolution, or they were extracted from solution and stored for later ex situ analysis.

### Optical Methods

A Keyence VHX‐7000 digital optical microscope was used to image crystal morphologies and observe patterns of PbSO_4_ precipitation on BaSO_4_ crystals. A 2500x objective lens provided high‐resolution images before and after the PbSO_4_ precipitation reaction with a field of view of 118.8 × 89.2 µm. Coaxial Lighting and a combination of High Dynamic Range and Multi‐Lighting features provided an improved view of crystal surface details. The Fine Depth Composition provided increased depth of field. BaSO_4_ crystals were embedded in a thin layer of adhesive (Crystalbond 509 in the case of Figure 5A,B or JB Weld Plastic Bonder in C,D of the same figure) in a clean watchglass. They were rinsed with DI water and dried with N_2_ gas for preliminary ex situ imaging. Precipitation for the images shown in this paper was then initiated by adding 250 µL of 200 µm Pb(NO_3_)_2_ via micropipette and next adding an equal volume of 200 mm H_2_SO_4_. A glass cover was placed overtop the crystals and solution and covered with Parafilm to prevent evaporation. Each watchglass was left to sit for 24 h. A schematic of the precipitation reaction setup is shown in Figure S12 (Supporting Information). For postimaging ex situ, the remainder of the solution was removed using Kimwipes, the watchglass was rinsed with DI water, and N_2_ gas was used to dry the BaSO_4_ crystals.

## Conflict of Interest

The authors declare no conflict of interest.

## Supporting information



Supporting Information

## Data Availability

The data that support the findings of this study are available from the corresponding author upon reasonable request.
