# Supplementary material for: Strontium‐Enriched Barite for Enhanced PbSO4 Nucleation in Lead‐Acid Batteries
Source: Small. 2025 Mar 3;21(13):2409902. doi: 10.1002/smll.202409902 (PMC11962690; doi:10.1002/smll.202409902)
Supplement: Supplementary file 1 — Supporting Information [file SMLL-21-2409902-s001.pdf]

NANO · MICRO
small

Supporting Information

for *Small*, DOI 10.1002/smll.202409902

Strontium-Enriched Barite for Enhanced $PbSO_4$ Nucleation in Lead-Acid Batteries

Colin T. Campbell, Ajay S. Karakoti, Shannon J. Lee, Carinna Lapson, David Reed, Benjamin A. Legg and Vijayakumar Murugesan**

Strontium-enriched $BaSO_4$ for Enhanced $PbSO_4$ Nucleation in Lead-acid Batteries

Colin T. Campbell, Ajay S. Karakoti, Shannon J. Lee, Carinna Lapson, David Reed, Benjamin A. Legg, Vijayakumar Murugesan**

E-mail: benjamin.legg@pnnl.gov, vijay@pnnl.gov

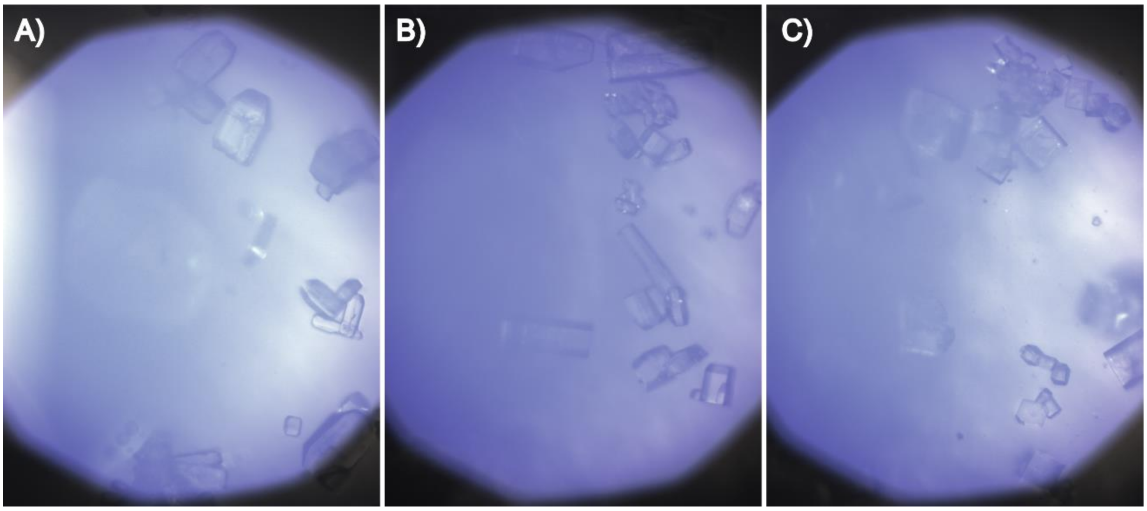

Figure S1. Shows a trio of in-situ optical microscopy images of barite crystals taken before AFM measurements **(A)** 0%Sr samples, **(B)** 6%Sr samples, and **(C)** 17%Sr samples. These illustrate the similar particle sizes obtained in each synthesis, and the variety of crystal morphologies present.

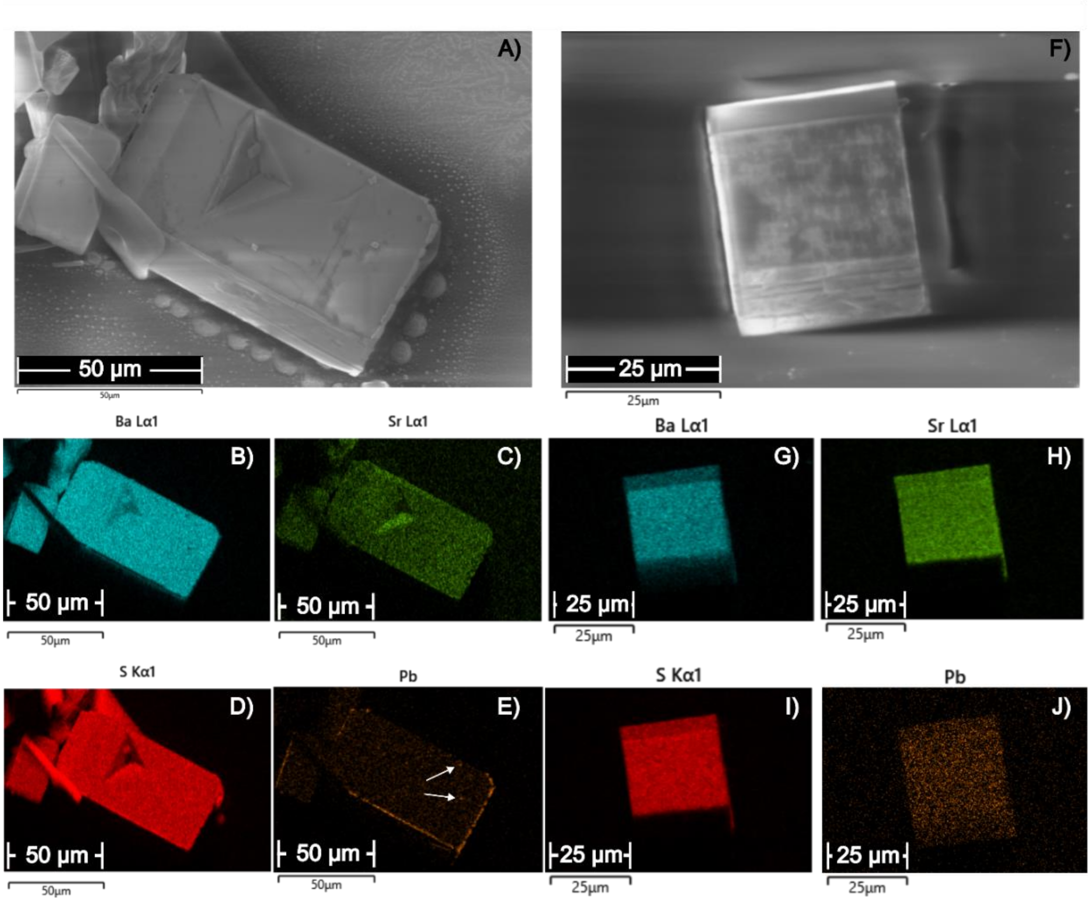

Figure S2. **(A)** Shows SEM image of a 6%Sr sample after Pb-exposure from in-situ AFM experiments. Corresponding EDS maps are shown for **(B)** barium, **(C)** strontium, **(D)** sulfur, and **(E)** lead. These Sr and Ba maps mirror each other, indicating uniform incorporation. The Pb map indicates significant $PbSO_4$ accumulation at sample edges, and from two growths occurring at the location of multi-step topographic features in the surface of the sample (see white arrows). **(F)** Shows SEM image of a 17%Sr sample (again, after exposure to Pb-rich solution). Corresponding EDS maps are shown for **(G)** Ba, **(H)** Sr, **(I)** S, and **(J)** Pb. A very low signal from Pb is consistent with findings of a thin (~ 200 nm or less) layer of lead, uniformly distributed across the surface.

The distribution of elements in synthesized barites and $PbSO_4$ growths was examined using Energy Dispersive X-ray Spectroscopy (EDS), analyzing the Ba $L\alpha_1$, Sr $L\alpha_1$, S $K\alpha_1$, and Pb $L\alpha$ lines. Figure S2 A-E shows a 6%Sr sample after the growth of $PbSO_4$ precipitates from in-situ experiments, with a generally uniform distribution of Sr throughout the sample (i.e. no evidence of zonation in these crystals). The lead signal is concentrated at sample edges, with distinct crystals highlighted in Figure S2 E. This is consistent with the formation of distinct crystals, localized on specific structures, in 6%Sr samples as observed in Figures 3-5. Figure S2 F-J shows a 17%Sr sample after the growth of PbSO4 precipitates (with uniform Sr distribution). Here, we see a very faint, uniform distribution of lead on the surface, with no evidence for preferential incorporation at edges, or in individual crystals. This is consistent with the formation of thin $PbSO_4$ films across the sample surface at 17%Sr, as in Figures 3-5.

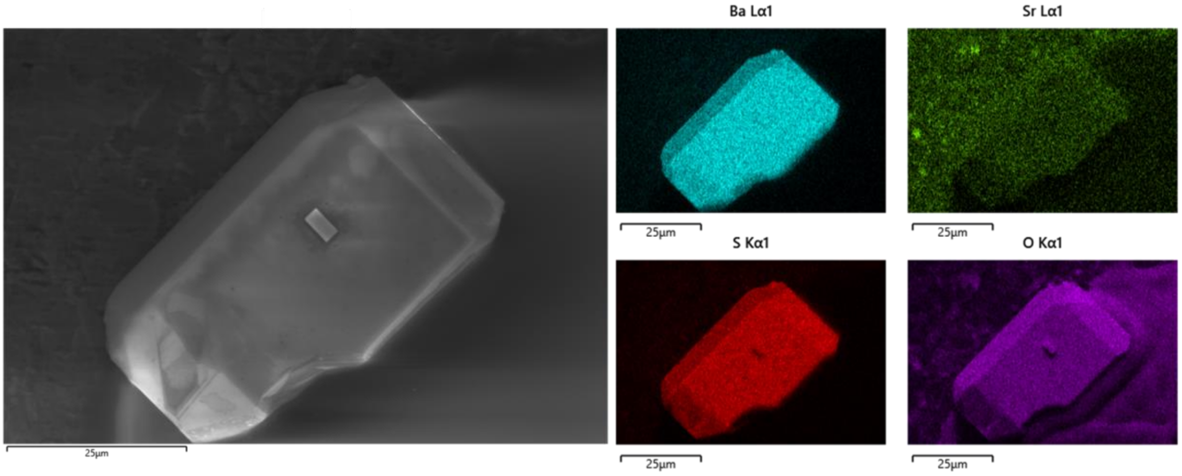

Figure S3. SEM and corresponding EDS maps of a pure barite particle (0%Sr) shows strong Ba, S, and O signals, but very little strontium (reflected by high noise in the normalized Sr EDS image, and very little signal relative to background).

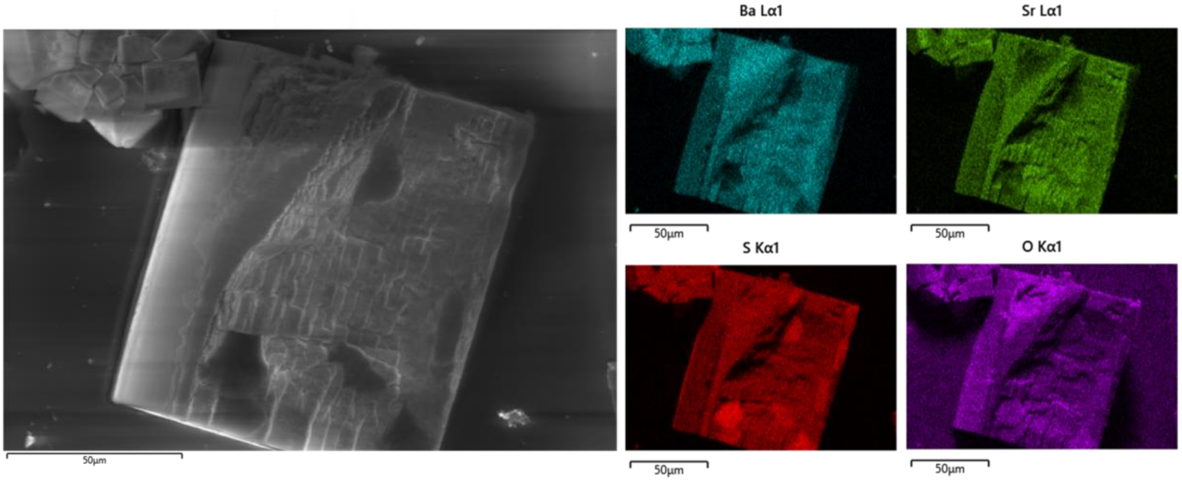

Figure S4. SEM and corresponding EDS maps of an additional 17%Sr particle shows both Ba and Sr distributed throughout the particle, although there are variations in intensity that are anti-correlated in some locations, which is an indicator for possible zonation., likely reflecting some zonation.

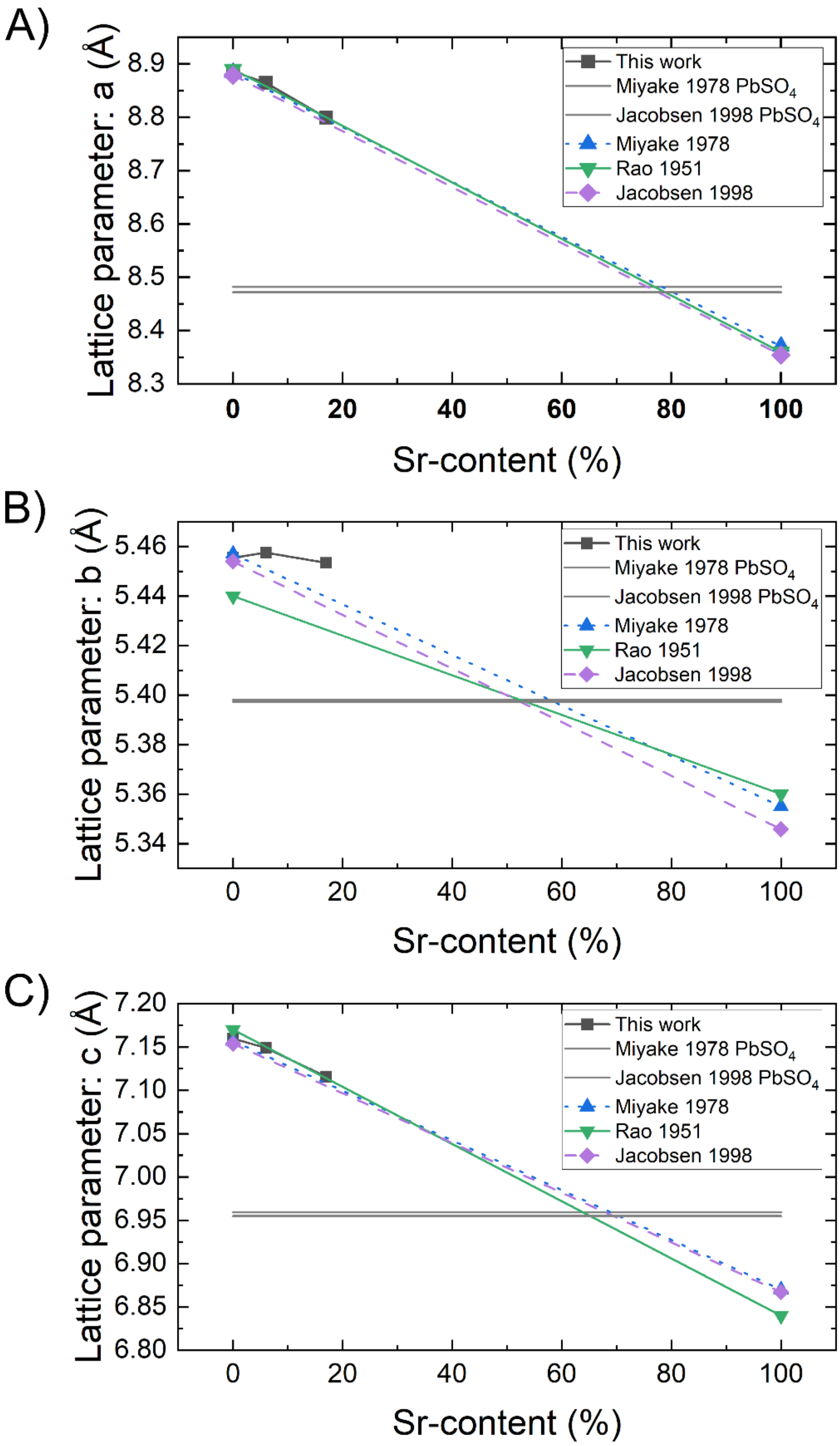

Figure S5 shows (A-C) the lattice parameters a, b, and c for $(Sr,Ba)SO_4$ samples as a function of strontium content, with comparison to several sources in literature.[1-3]

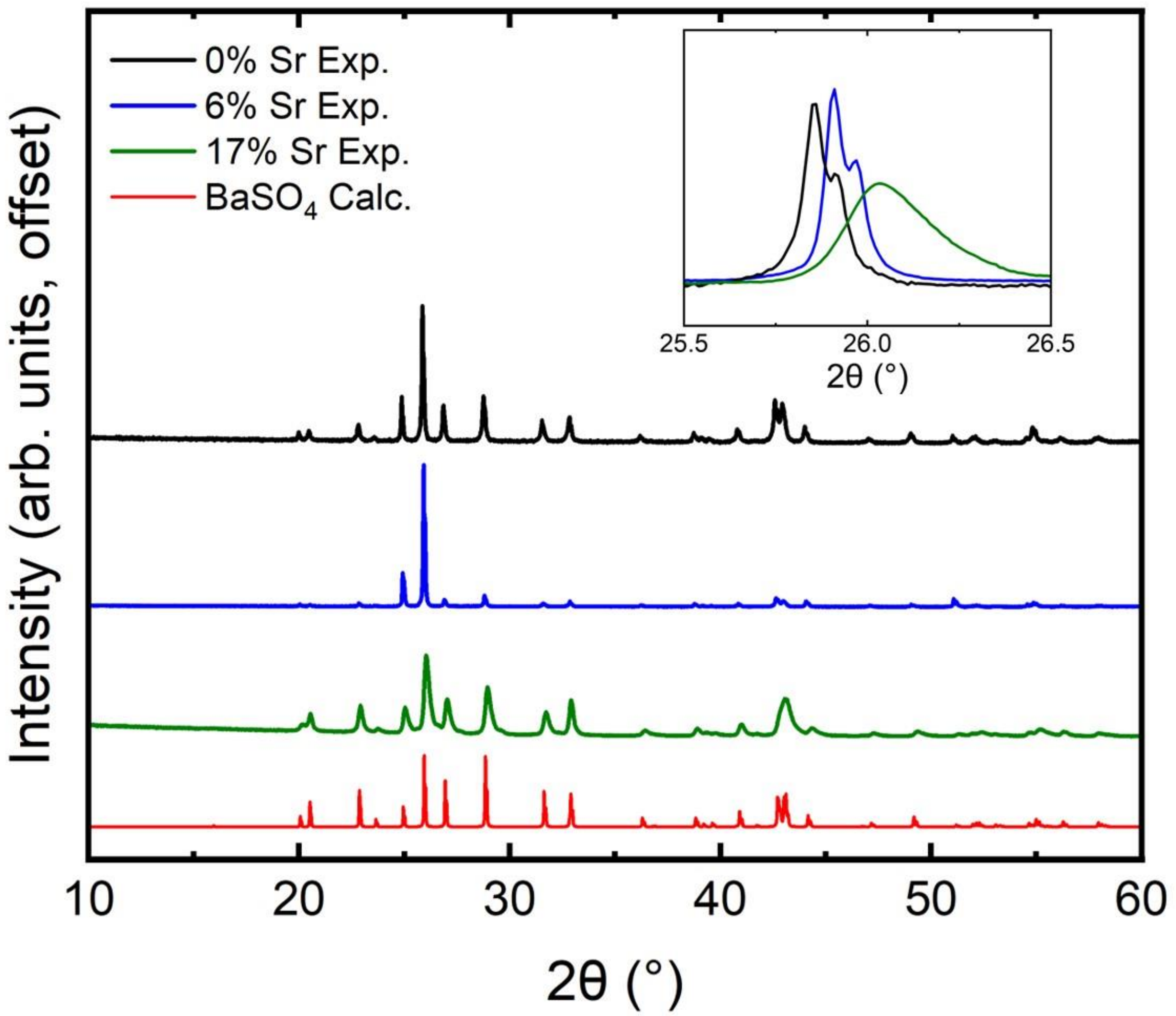

Figure S6 PXRD data obtained using Rigaku Miniflex 6G benchtop X-ray diffractometer. Patterns agree well with predicted barite pattern, and there are no signs of impurity phases. Peak shifts for 6%Sr and 17%Sr relative to 0%Sr show contractions in the lattice parameter with addition of Sr. Significant peak broading for 17%Sr is indicative of microstrain and local concentration variations (i.e. zonation), but there are no signs of peak splitting that might indicate a bimodal distribution of Sr-rich and Sr-poor particles.

Table S1. Mass Spectrometry of (Ba,Sr)SO_4 particle dissolution into 4.5 M H_2SO_4. Suspensions were prepared by adding between 37 and 40 mg into 21 mL of 4.5 M H_2SO_4 at 50 ^oC. All measuremements were performed in duplicate. RSD is estimated relative standard deviation.

Condition	Ba (mg/L)		Sr (mg/L)	
	Value	RSD	Value	RSD
17%Sr, Total added in solid	962	-	125	-
17%Sr, Dissolved for 90 h	0.004	47.8	1.56	2.5
17%Sr, Dissolved for 180 h	0.008	10.7	1.58	0.9
6%Sr. Total added in solid	980	-	40	-
6%Sr, Dissolved for 90 h	0.02	4.9	0.06	1.7
6%Sr, Dissolved for 180 h	0.03	13.5	0.13	0.1
0%Sr, Total added in solid	1036	-	-	-
0%Sr, Dissolved for 90 h	0.012	8.4	0.002	23.3
0%Sr, Dissolved for 180 h	0.014	7.6	0.12	15.9
H_2SO_4 Blank (No Barite)	0.004	32.4	0.005	8.2

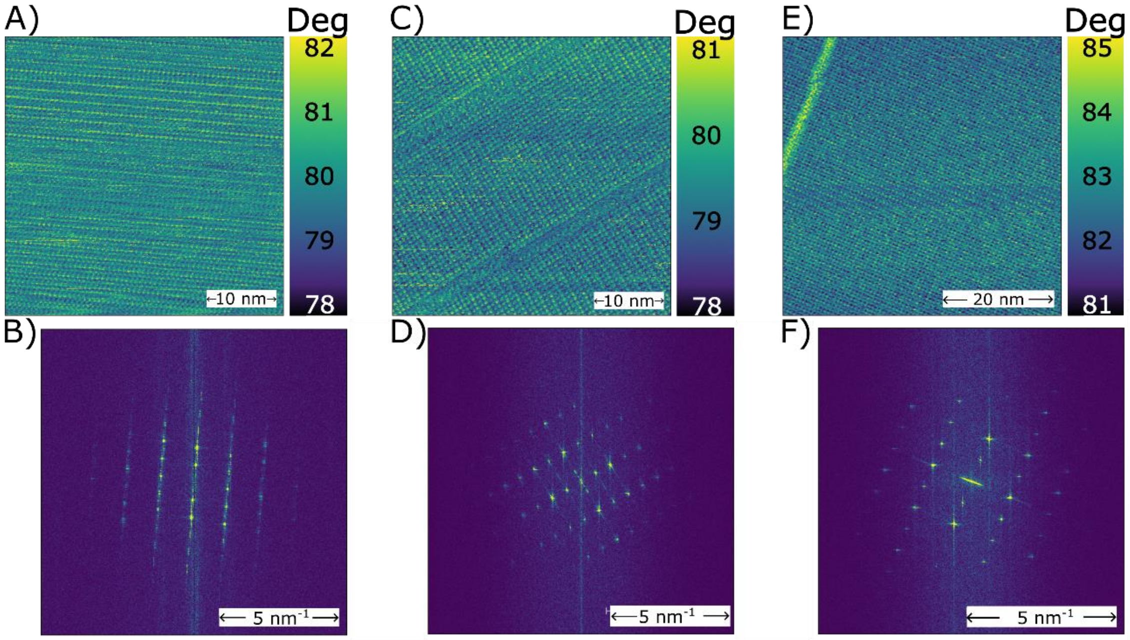

Figure S7. (A) Shows high resolution AFM phase-image of a 0%Sr surface, and **(B)** shows corresponding Fast Fourier Transform (FFT), **(C)** shows high-resolution AFM phase-image of a 6%Sr surface and **(D)** shows corresponding FFT. **(E)** shows high-resolution AFM phase-image of 17%Sr surface and **(F)** shows corresponding FFT. All were obtained in-situ, but prior to exposure to Pb-containing solution. The patterns all show a roughly rectangular lattice, with orthogonal lattice vectors of approximately 0.7 and 1.4 nm in length, which correspond to the known [001] and [$1\bar{2}0$] vectors on the $BaSO_4$ (210) surface. Fourier transform and lattice vector measurement was performed using the "Gwyddion" software package.[4]

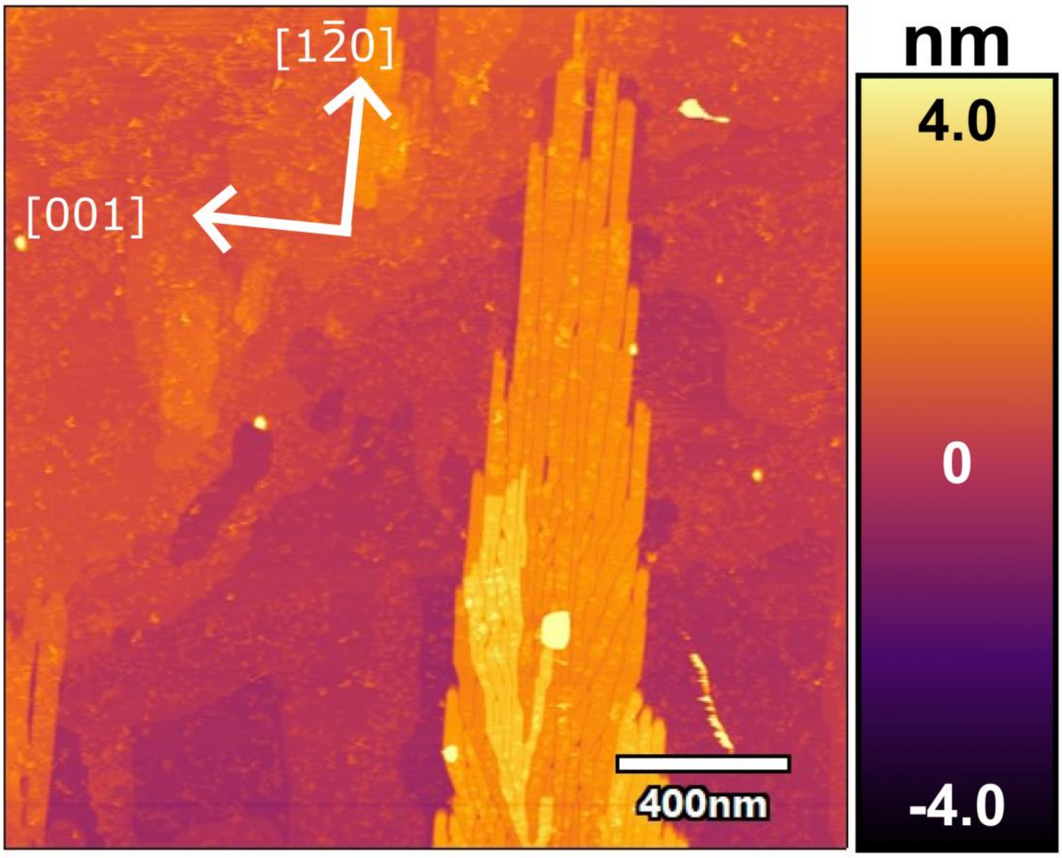

Figure S8. Shows the topography in the area around an epitaxial transient feature on the Sr-free (210) $BaSO_4$ surface after 15 minutes of exposure to flowing 50 µM $Pb(NO_3)_2$, 100 mM H_2SO_4 solution. (Image obtained ~8 minutes after Figure 3 B). The epitaxial transients consist of elongated branches, packed side-by-side, and growing preferentially in the [1$\bar{2}$0] direction. They occasional diverting along a diagonal direction when they are unable to grow in a straight line along the [1$\bar{2}$0] direction. The branches range in width from 20 nm to almost 100 nm across.

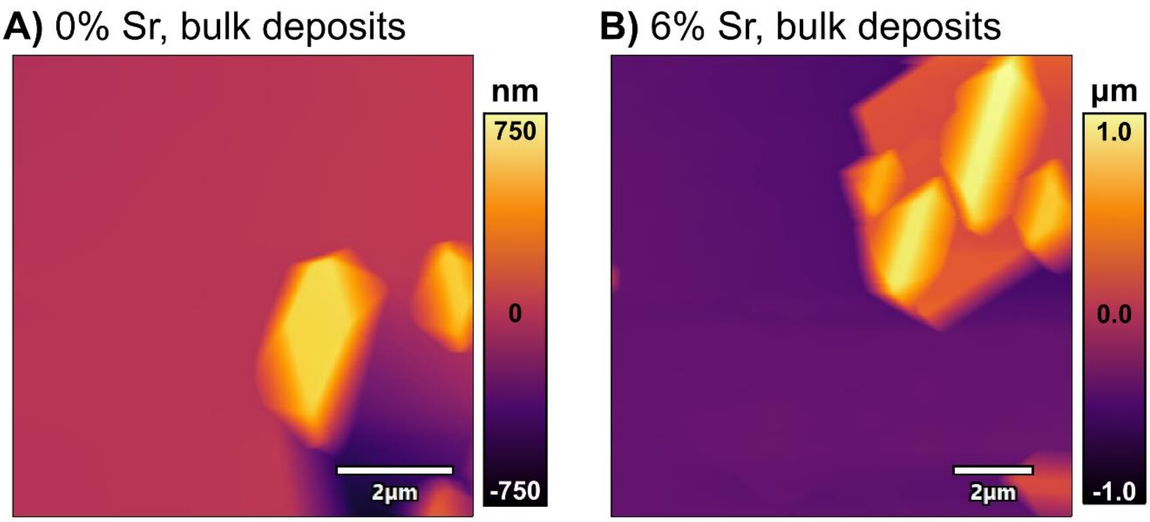

Figure S9. Shows topography images of bulk, euhedral $PbSO_4$ crystals formed on the (001) $BaSO_4$ surface. **(A)** On a 0%Sr sample. **(B)** On a 6%Sr sample. These images are taken from the same samples shown in Figure 4 A - F, after 50 minutes of flow of 50 µM $Pb(NO_3)_2$, 100 mM H_2SO_4 solution. In both cases, the euhedral crystals appear to have formed at the edge of topographic features that are hundreds of nanometers tall.

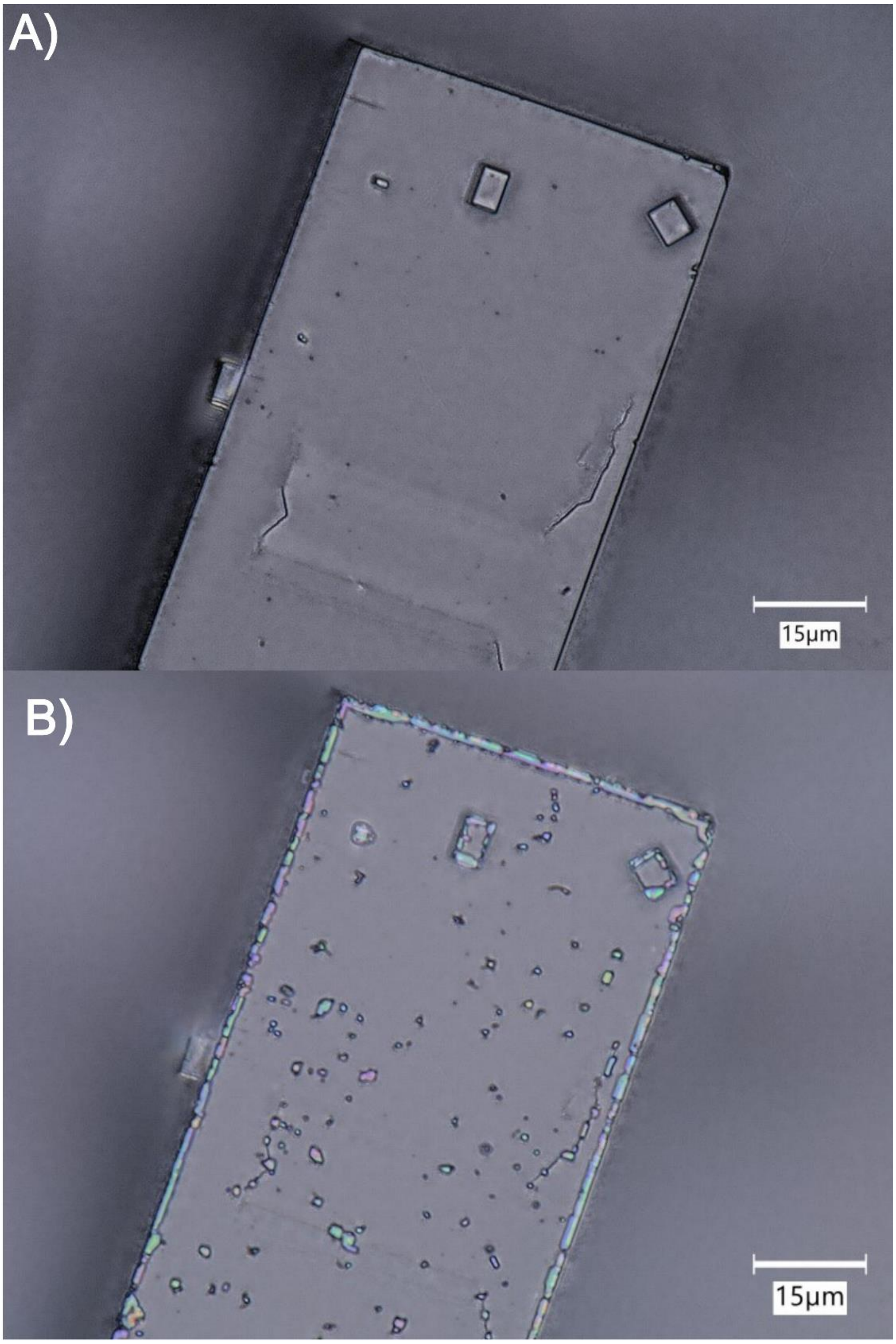

Figure S10. Shows examples of ex-situ optical images of a 6%Sr crystal **(A)** before, and **(B)** after 24 hours exposure to a supersaturated solution of 100 μM $Pb(NO_3)_2$, 100 mM H_2SO_4. Like figure 5 B), this shows sparse nuclei on the surface and a significant preference for step edges, consistent with the in-situ observations of preferential nucleation and growth of $PbSO_4$ at step edges and hillocks seen in the 0%Sr and 6%Sr AFM experiments.

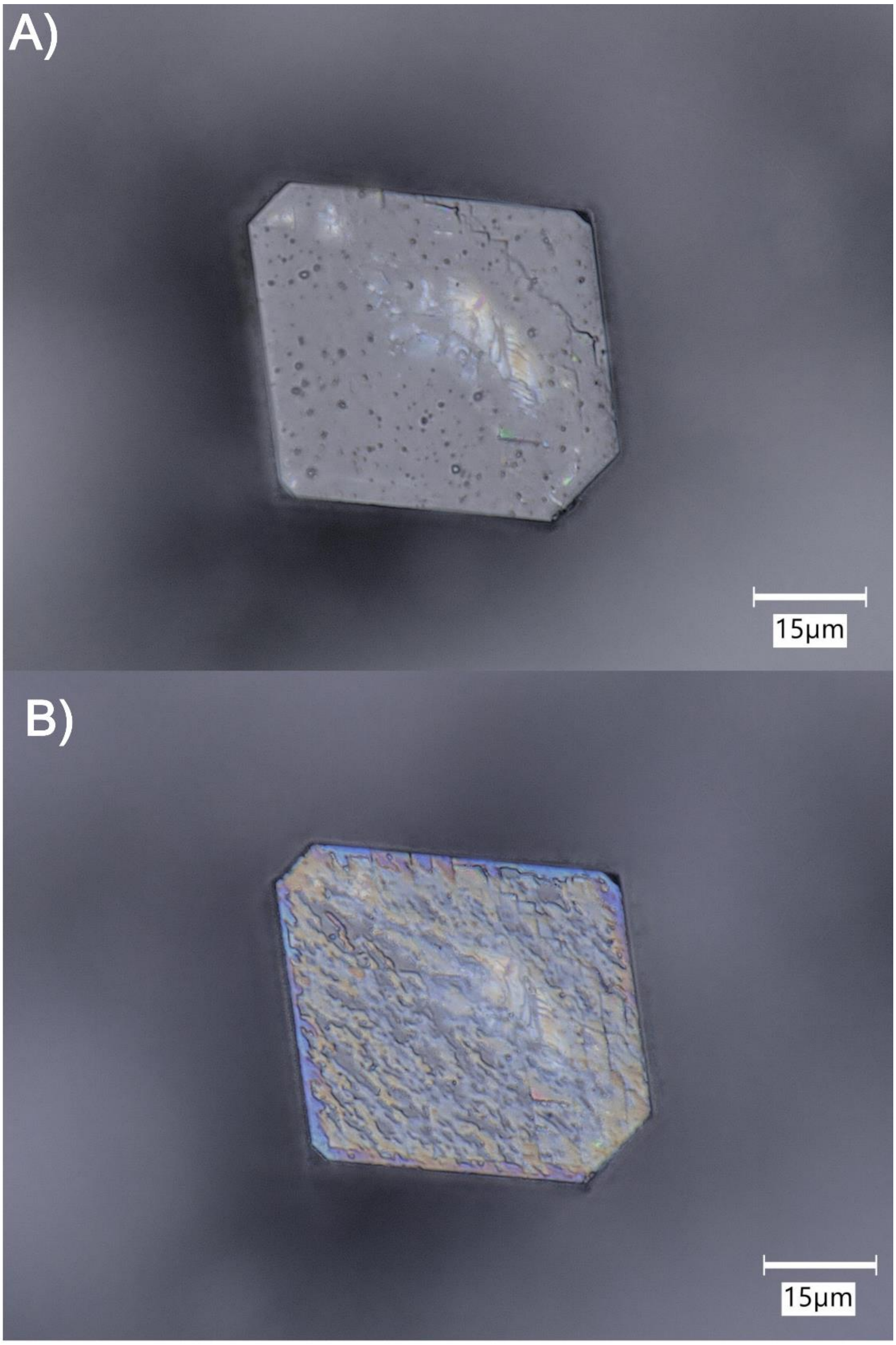

Figure S11. Shows examples of ex-situ optical images of a 17%Sr crystal **(A)** before, and **(B)** after 24 hours exposure to a supersaturated solution of 100 μM $Pb(NO_3)_2$, 100 mM H_2SO_4. As in Figure 5, an iridescent pattern of color appears on the surface after exposure, indicating films on the order of hundreds of nanometers in thickness.

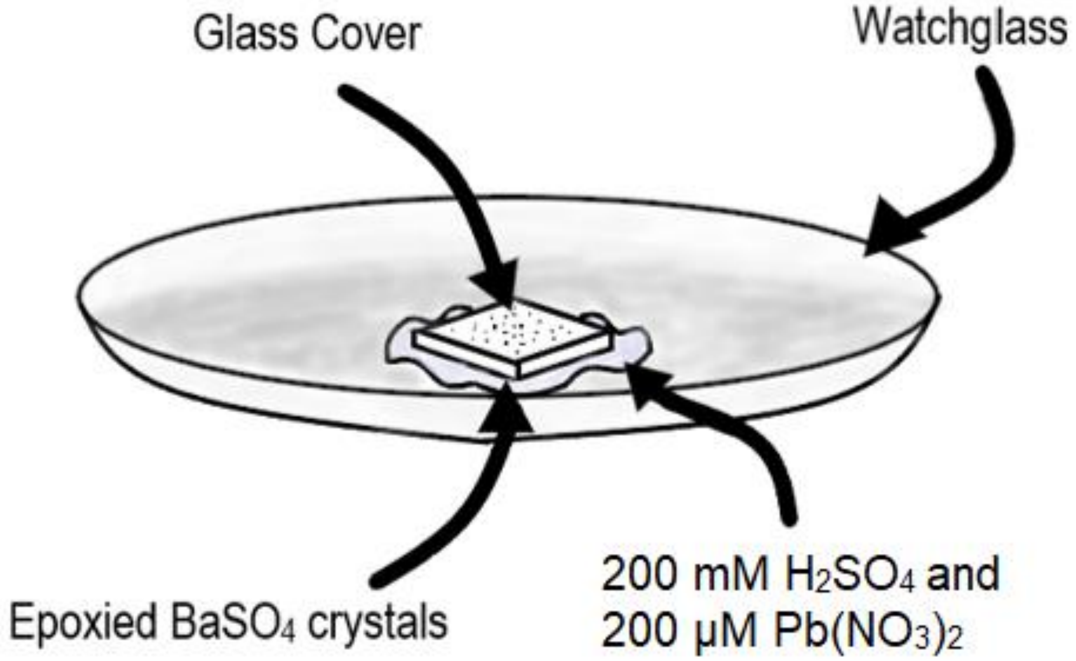

Figure S12. Schematic detailing the ex-situ optical microscopy exposure protocol.

References:

(1) M. Miyake, I. Minato, H. Morikawa, S.-I. Iwai, Am. Mineral. 1978, 63, 506.

(2) T. S. Rao. Proc. Indian Acad. Sci. (Math. Sci.) 1951, 33, 251.

(3) S. D. Jacobsen, S. R. Joseph, R. J. Swope, R. T. Downs, Can. Mineral. 1998, 36, 1053.

(4) D. Nečas, P. Klapetek, Open Phys. 2012, 10, 181.
